# 浅析MIDD在抗肿瘤药物临床研究中的应用

**DOI:** 10.3779/j.issn.1009-3419.2022.101.38

**Published:** 2022-07-20

**Authors:** 孟洋 余, 洪允 王

**Affiliations:** 100730 北京，中国医学科学院北京协和医学院，北京协和医院，临床药理研究中心 Clinical Pharmacology Research Center, Peking Union Medical College Hospital, Peking Union Medical College and Chinese Academy of Medical Sciences, Beijing 100730, China

**Keywords:** 模型引导的药物开发, 暴露量-效应关系, 群体药代动力学, 奥希替尼, 帕博利珠单抗, Model-informed drug development, Exposure-response relationship, Population pharmacokinetics, Osimertinib, Pembrolizumab

## Abstract

抗肿瘤药物是新药研发的热点方向，其临床研究具有研发周期长、研发成本和风险高的特点。模型引导的药物开发（model-informed drug development, MIDD）通过建模与模拟，能够对生理学、药理学以及疾病进程等信息进行整合和定量分析，降低药物研发成本，提高临床研究效率。本文以奥希替尼和帕博利珠单抗为例，以问题为导向，阐述MIDD在抗肿瘤药物临床研究各个阶段的具体应用，旨在为MIDD指导抗肿瘤临药物床研究提供一定的借鉴和参考。

## Abstract (en)

The antitumor drug has become one of the focused areas in new drug research and development. Their clinical research generally consumes a long period of time, with high cost and high risk. Model-informed drug development (MIDD) integrates and quantitatively analyzes physiological, pharmacological, and disease progression information through modeling and simulation, which can reduce the cost of drug development and improve the efficiency of clinical research. In this essay, Osimertinib and Pembrolizumab are given as examples to illustrate the specific application of MIDD in different phases of clinical research, aiming to provide references for the application of MIDD to guide the clinical research of antitumor drugs.

## 1 引言

中国国家癌症中心统计数据^[1]^显示，2016年我国共新发约406.4万恶性肿瘤病例，死亡病例约241.35万。其中，肺癌、肝癌、胃癌、结直肠癌和食管癌位居癌症死亡病因前五位，占癌症死亡总人数的69.3%，癌症新发病例和死亡病例数均高于往年。预计未来十年，中国的恶性肿瘤负担将继续加重。药物治疗是肿瘤治疗的重要手段，抗肿瘤药物是当前国际以及国内新药研发的热点。据报道，2020年我国抗肿瘤药物临床试验占全年药物临床试验总量的42.6%^[2]^。由于肿瘤是危及生命的严重疾病，患者往往存在明显的未被满足的临床需求，因此长期以来抗肿瘤药物的临床研究具有其特殊性和迫切性。2021年11月，国家药监局药品审评中心颁布了《以临床价值为导向的抗肿瘤药物临床研发指导原则》，对创新药研发提出了更高的要求，明确了以临床价值为导向、以患者为核心的研发理念^[3]^。众所周知，临床试验设计是决定研发成功与否的重要因素之一，良好的试验设计不仅有助于达到试验目的，同时还能提高研发效率。

模型引导的药物开发（model-informed drug development, MIDD）采用数学方法，基于统计学原理，通过模型模拟对生理学、药理学以及疾病过程等信息进行整合和定量分析，最终实现指导新药临床试验设计和分析临床试验数据等^[4]^。MIDD的常用方法包括群体药代动力学模型（population pharmacokinetics, PopPK）、药代动力学/药效动力学模型（pharmacokinetics/pharmacodynamics, PK/PD）、暴露量-效应（exposure-response, E-R）关系模型、基于生理的药代动力学模型（physiologically based pharmacokinetics, PBPK）等。在抗肿瘤药物的临床研究过程中，采用模型引导的药物开发方法能够在一定程度上优化临床试验设计、降低药物研发成本及风险，加速临床试验进程，支持药品注册申报。本文将以近年两个代表性的抗肿瘤药物奥希替尼和帕博利珠单抗为例，分别从靶向药物和肿瘤免疫药物角度论述MIDD在抗肿瘤药物临床研究中的具体应用。

## 2 MIDD在奥希替尼临床研究中的应用

奥希替尼（Osimertinib）是第三代不可逆表皮生长因子受体（epidermal growth factor receptor, EGFR）酪氨酸激酶抑制剂（tyrosine kinase inhibitor, TKI）。与第一、二代EGFR-TKI相比，奥希替尼可选择性抑制*EGFR*敏感性突变和*EGFR* T790M耐药性突变，同时保留野生型EGFR^[5]^，临床上用于治疗非小细胞肺癌（non-small cell lung cancer, NSCLC），并已被证实对发生中枢神经系统转移的NSCLC患者有效^[6, 7]^。作为第三代TKI靶向药物，从临床研究到获批上市，奥希替尼仅用了短短两年时间，是美国食品药品监督管理局（Food and Drug Administration, FDA）有史以来最快批准上市的抗癌药物，成为该领域延续至今的一个新药开发传奇。在奥希替尼的临床研究过程中，MIDD的应用在预测药物-药物相互作用（drug-drug interaction, DDI）及不同人群用药方案外推方面均发挥了重要作用。

预测DDI方面，基于临床前研究结果：奥希替尼主要由肝脏代谢，经细胞色素P450 CYP3A4/5酶的代谢分数约为54%，肾脏清除所占比重很小，体外转运体实验数据显示，奥希替尼为乳腺癌耐药蛋白（breast cancer resist-ance protein, BCRP）抑制剂^[8]^，这提示当该药物与CYP3A酶底物、诱导剂、抑制剂或BCRP底物联用时，可能发生DDI，由此改变药物在体内的药物暴露，进而影响疗效和引发安全性问题。因此，研究团队开展了奥希替尼与伊曲康唑（CYP3A强抑制剂）、利福平（CYP3A强诱导剂）、辛伐他汀（CYP3A底物）及瑞舒伐他汀（BCRP底物）的DDI研究^[9, 10]^。在上述临床研究的基础上，Pilla等^[11]^使用Simcyp软件（该软件是被主要监管机构、工业界和学术界普遍认可的PBPK建模和模拟平台），结合奥西替尼体外酶学实验数据，建立验证奥希替尼的PBPK-DDI模型，并根据临床用药需求，前瞻性模拟和预测奥希替尼与依非韦伦（CYP3A中等诱导剂）、地塞米松（CYP3A弱诱导剂）的潜在相互作用。预测结果显示，80 mg奥希替尼与600 mg依非韦伦合用时，峰浓度（C_max_）值降低了42%，药物浓度-时间曲线下面积（area under the curve, AUC）降低了36%；与8 mg地塞米松合用时，未发现其对奥希替尼AUC的诱导作用，C_max_仅降低了1%。由于奥希替尼具有较宽的治疗窗，在20 mg-240 mg剂量范围内开展的AURA I期临床试验尚未达到最大耐受剂量^[12]^，基于E-R关系分析，认为2倍以内的奥希替尼暴露变化对其风险获益比影响不大。因此，判断奥西替尼与上述药物合用时，无需进行剂量调整。与此同时，研究人员使用该模型对奥希替尼与利福平（强CYP3A诱导剂）联用时的剂量调整进行了评估。结果显示，当与利福平合用时，需将奥希替尼剂量调整为160 mg，才能达到与单独使用80 mg奥希替尼情况下相似的暴露。上述基于PBPK模型的DDI预测和模拟研究策略，充分利用体外和部分临床试验数据，豁免了非必要临床试验，节省了研究成本并加速了研发进程，是MIDD在创新药临床开发的代表性案例。目前，我国国家药品监督管理局（National Medical Products Administration, NMPA）及美国FDA、欧洲药品管理局（European Medicines Agency, EMA）、日本药品和医疗器械综合管理局（Pharmaceuticals and Medical Devices Agency, PMDA）等各国药政管理部门已经普遍认可和接受PBPK模型用于支持DDI临床研究。我国药品审评中心在2021年1月颁布的《药物相互作用研究技术指导原则（试行）》中明确指出：“新药临床研发中，需要对DDI研究做出预测以辅助临床试验设计，有时也可以根据预测结果评价临床药物相互作用”，建议采用经验证的PBPK模型模拟和支持DDI临床试验设计^[13]^。

在药物临床开发阶段，根据研究目标，无论是小样本量的早期探索性临床研究，还是大样本量的确证性临床研究，往往都需要在符合入选/排除标准的“特定”人群中开展，不可避免地会导致无法充分评估患者多样性对于药物的影响。应用MIDD方法，能够基于现有的临床试验数据，对药物在不同人群的用药方案进行合理外推。在奥西替尼的临床研究过程中，为确定该药物在广泛人群中的用药方案，Brown等^[14]^建立了奥希替尼及其活性代谢产物AZ5104的群体药代动力学模型，纳入了两项在NSCLC患者（*n*=748）中开展的临床试验及一项在健康受试者（*n*=32）中开展的临床试验数据，评估了年龄、性别、剂型、吸烟状况及肝肾功能等多种因素对于药物的影响，并以客观缓解率、缓解持续时间、目标病灶较基线变化百分比为主要疗效指标，以皮疹、腹泻、QTcF为主要安全性指标进行了E-R关系研究。协变量筛选结果表明，在43 kg-90 kg体重范围内，奥希替尼稳态血药浓度-时间曲线下面积（area under the steady-state concentration-time curve, AUCss）与中位体重（62 kg）的AUCss相比有-20%到+30%的变化，其代谢物AZ5104的AUCss变化范围为-40%到+50%；活性代谢物AZ5104的AUCss在非高加索患者中出现了10%-23%的降低；肝功能损伤标志物血清白蛋白水平对奥希替尼的分布容积存在影响而对AUCss无影响；年龄、性别、剂型、吸烟状况及肝肾功能对奥希替尼和AZ5104的PK均无影响。最终经过群体模型的评估，确定体重、血清白蛋白和种族等3个变量对PK存在显著影响。在此基础上，研究人员通过E-R分析，进一步评估了上述变量引发的PK暴露变化是否会影响药物的疗效和安全性。结果显示，上述协变量（体重、血清白蛋白和种族）虽然会在一定程度上改变奥西替尼的体内药动学行为，但均不会引起药物暴露量增加2倍或降低到50%以下，综合分析后认为这些变化不具备临床相关意义，因此无需根据体重、血清白蛋白水平和种族对80 mg *qd*给药进行剂量调整。E-R关系分析进一步发现，患者出现皮疹或腹泻的概率随着奥希替尼暴露量增加而升高；ΔQTcF与奥希替尼浓度间存在线性关系（*P* < 0.000, 1），奥希替尼浓度每增加10 nmol/L，ΔQTcF的平均增量为0.271 ms（95%CI: 0.241-0.301）；在研究的剂量范围内，未发现奥希替尼暴露量与疗效学指标间的关系。

上述MIDD研究，采用PopPK模型和E-R分析的建模与模拟技术，基于既往临床试验数据，充分评估了各种临床实践中复杂因素对于药物疗效和安全性的影响，阐明了药物的获益-风险关系，支持了在美国、欧盟及日本获批的80 mg *qd*给药方案，能最大限度地提高临床疗效，同时最小化不同人群中基于协变量的不良反应的概率。

## 3 MIDD在帕博利珠单抗临床研究中的应用

帕博利珠单抗（Pembrolizumab）是一种针对PD-1受体的高选择性IgG4-kappa人源化单克隆抗体，通过将小鼠高亲和力抗人PD-1抗体的可变区域序列移植到含有稳定S228P Fc突变的人IgG4-kappa同种型框架上而产生^[15]^，能够有效阻断T细胞表面PD-1受体与肿瘤细胞PD-L1、PD-L2配体结合，重新恢复机体的抗肿瘤免疫功能。帕博利珠单抗是FDA历史上首个被纳入突破性治疗（breakthrough therapy designation, BTD）审评程序的抗肿瘤药物，是全球首个批准上市的PD-1抑制剂^[16]^。从临床前研究到提交新药临床试验申请（investigational new drug, IND），从首次人体试验（first in human, FIH）到队列拓展试验，从提交新药申请（new drug application, NDA）到上市后剂量优化，MIDD贯穿了帕博利珠单抗临床研究的全生命周期，并在其剂量选择及给药方案优化中发挥了极其重要的支持作用。

作为PD-1抑制剂类药物，帕博利珠单抗具有较为明确的疗效指标，即炎性细胞因子释放量与药效呈正相关性^[17]^，因此，在FIH早期临床开发阶段，研究人员通过在健康受试者及部分肿瘤患者外周血中开展的体外白细胞介素-2（interleukin-2, IL-2）刺激试验评估了帕博利珠单抗调节T细胞活性的能力，获得了药物浓度与IL-2释放的E-R反应关系，通过建立Imax模型，初步估测出1 mg/kg给药即可达到体外靶标受体占据饱和^[18]^，从而确定了FIH试验的最大推荐起始剂量，启动了在晚期实体瘤患者中开展的KEYNOTE‐001研究。该研究的FIH剂量递增部分对帕博利珠单抗1 mg/kg、3 mg/kg、10 mg/kg *q2w*给药和2 mg/kg、10 mg/kg *q3w*给药进行了评估，在各剂量组中均观察到抗肿瘤活性且未观察到剂量限制性毒性，总体安全性可控^[19]^。

为了能够进一步在更大的患者群体中选择最佳疗效剂量以开展试验，建模和模拟在指导KEYNOTE-001扩展队列的研究设计中继续发挥了关键性的作用。Lindauer等^[20]^利用临床前小鼠实验数据构建了帕博利珠单抗PK/PD肿瘤生长抑制模型并外推至人类，预测药物对人体肿瘤的抑制率。结果表明：当剂量为2 mg/kg *q3w*时，帕博利珠单抗的受体占据率超过95%，实现肿瘤体积减少大于30%的概率达到坪值（图 1），提示2 mg/kg *q3w*可作为临床试验的有效剂量。与此同时，Elassaiss‐Schaap等^[21]^基于FIH试验数据，通过“学习与确认”（learn and confirm）循环进行建模与模拟，并随着研发过程的推进对模型进行不断更新和完善，揭示了KEYNOTE-001试验中帕博利珠单抗的PK/PD关系。其中，血液IL-2刺激率反映了帕博利珠单抗与靶标的结合情况，被作为抗肿瘤疗效标志物（即PD指标）。模拟结果显示，当给药剂量低于1 mg/kg时，达到目标受体占据率的可能性显著降低（图 2A），而2 mg/kg *q3w*或更高剂量给药时，有90%及以上的概率实现至少95%的靶标占据率（图 2B）。基于此模拟结果，研究人员推荐2 mg/kg作为临床最佳疗效剂量。在后续临床研究中，该剂量水平（2 mg/kg）被用于在晚期黑色素瘤和NSCLC患者中开展的更大规模的随机对照试验^[22, 23]^，成功表征了帕博利珠单抗的临床获益与风险，即在晚期黑色素瘤和NSCLC患者的临床试验中，该剂量选择的合理性在临床疗效和安全性评估中得以充分验证。

**图 1:**
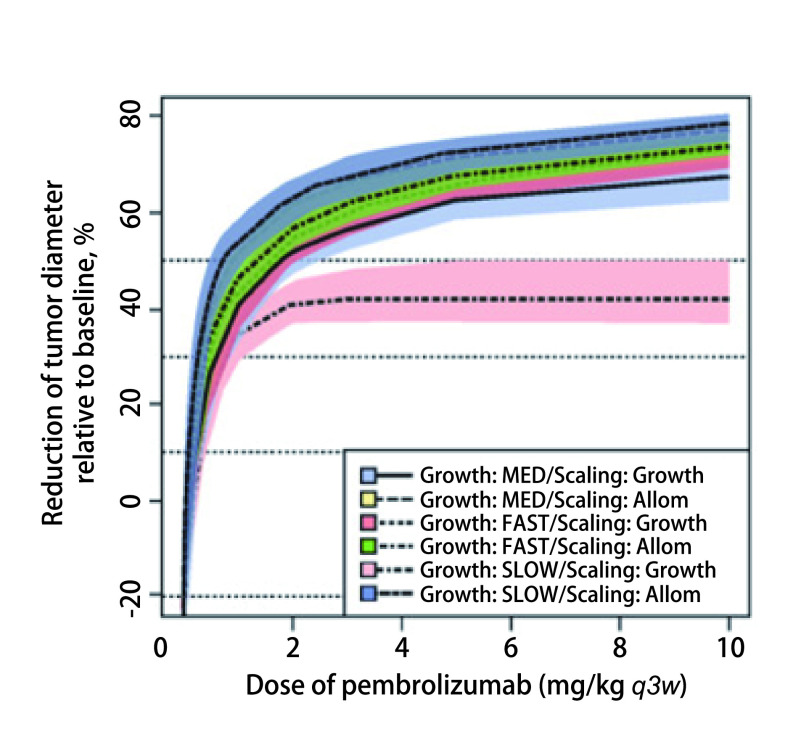
模拟快速（FAST）、中等（MED）和慢速（SLOW）生长速率的6种黑色素瘤在6个月内使用帕博利珠单抗*q3w*治疗后的肿瘤反应（相对于基线直径的变化百分比）。图已从Lindauer A获得CC BY-NC-ND 4.0许可证。 Simulated tumor response in melanoma (percentage change from baseline diameter) following treatment with pembrolizumab (once every 3 weeks) over 6 months for the six scenarios for melanoma using the fast, medium (MED), and slow growth rates^[20]^. Figure available from Lindauer A under CC BY-NC-ND 4.0 license.

**图 2:**
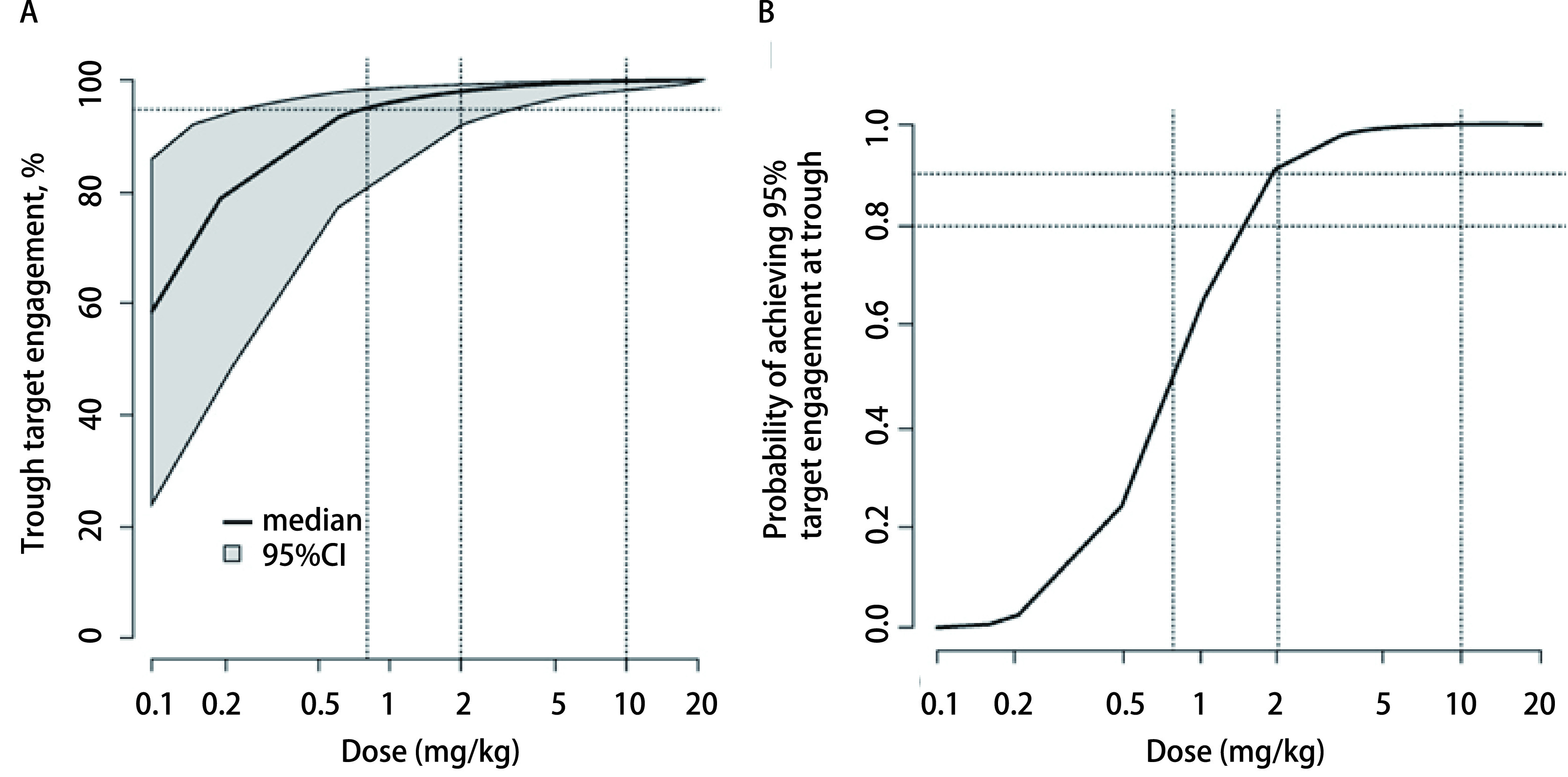
受体占据率模拟结果 Simulations of target engagement

(A) 模拟不同给药剂量（*q3w*）达稳后帕博利珠单抗的受体占据率及其95%置信区间。(B) 不同剂量*q3w*给药后在谷浓度达到95%受体占据率的概率（模拟人群百分比）。图已从Elassaiss Schaap J获得CC BY-NC-ND 4.0许可证。

(A) Percentage of target engagement with a band denoting the 95% confidence interval (95%CI) for an every-3-weeks dosing regimen. (B) Probability (percentage of subjects within a simulated population) of achieving 95% target engagement at trough for different doses given every 3 weeks^[21]^. Figure available from Elassaiss-Schaap J under CC BY-NC-ND 4.0 license.

随后，为确定人口统计学因素（年龄、身高和体重）、病理生理学和疾病等各种因素对帕博利珠单抗PK行为的影响，以支持该药在更广泛人群临床治疗中的合理应用，研究人员纳入了KEYNOTE-001、KEYNOTE-002和KEYNOTE-006研究的汇总数据，对从晚期黑色素瘤、NSCLC和其他实体肿瘤类型的患者中获得的稀疏采样数据进行了PopPK模型分析。结果表明，帕博丽珠单抗的PK曲线与经典治疗性单克隆抗体一致，表现出有限的分布容积、低清除率和低变异性，内在因素（如：体重、年龄、性别、肿瘤类型及负荷、肾肝功能损伤）和外在因素（如：伴随用药）对帕博利珠单抗的暴露没有具有临床意义的影响。该研究结果支持了在不同的患者亚群中使用批准的2 mg/kg *q3w*剂量给药，而无需进行任何调整^[26]^，减少了临床用药的不便及可能出现的用药混乱。随后，Freshwater等^[27]^在上述模型工作基础上，进一步探讨了固定剂量给药（200 mg *q3w*）的可行性。KEYNOTE-10、KEYNOTE-055、KEYNOTE-024、KEYNOTE-164、KEYNOTE-045和KEYNOTE-052是后续在不同瘤种患者中开展的系列试验，研究人员将来自这些试验的血药浓度数据纳入模型并重新拟合，模拟了固定剂量给药（200 mg *q3w*）的系统暴露情况，并与基于体重给药（2 mg/kg *q3w*）的方案进行了对比。结果表明，200 mg *q3w*剂量的暴露分布与2 mg/kg *q3w*剂量有很大重叠，两种给药方案的PK变异性相当。此外，使用200 mg *q3w*固定剂量给药的临床试验的PK实测值数据进一步证实了基于PopPK模型预测的该方案的暴露的准确性，最终FDA批准了帕博利珠单抗由基于体重给药改为固定剂量给药（200 mg *q3w*）的申请^[28]^。

在帕博利珠单抗临床开发过程中，MIDD发挥了极其重要的作用。采用转化PK/PD模型、肿瘤生长抑制模型、群体PK模型等多种建模和模拟方法，在IND阶段支持FIH起始剂量的设置和方案设计；在队列拓展阶段，基于前期临床PK和PD数据，提出给药方案建议；在NDA阶段，评估各种协变量因素对于药物暴露的影响，以及是否可进行固定剂量给药等，阐明了药物的获益-风险关系。可以说，秉承以临床核心问题为导向研究策略，MIDD贯穿了帕博利珠单抗临床开发的全生命周期，成功支持了该药物在短时间内实现快速上市，为同类药物的临床开发提供了有价值的参考。

## 4 结语

抗肿瘤药物研发周期长、研发成本高，由于肿瘤疾病病理生理学的复杂性，患者群体往往异质性较大，开展临床研究的不确定性因素较多。因此，科学灵活地设计并开展临床试验尤为重要。MIDD方法在抗肿瘤药物临床研究中展现出的优势包括：①在早期临床开发阶段，通过模型外推法，基于非临床研究数据预测首次人体试验剂量，能够尽可能减少受试者（肿瘤患者）在无效剂量下的暴露，降低不必要的风险；②在探索性临床研究阶段，利用PK/PD模型整合前期临床试验数据并进行不同给药方案的模拟，探索剂量-暴露-效应关系，能够为后续临床研究筛选出既能充分保障受试者安全又能最大限度保留疗效获益的推荐剂量；③在上市后研究阶段，借助PopPK方法，可充分整合既往临床研究中的密集/稀疏采样数据，对影响药物PK、PD行为的内部、外部因素加以识别，为药物在患者亚群中用法用量的调整等提供支持。不难看出，在抗肿瘤药物临床研究中应用MIDD方法可以极大提高临床试验的质量与效率，降低临床开发过程的不确定性与成本。目前，美国FDA、EMA及PMDA等主要国家和地区的药品监管机构都已积极将MIDD方法应用于监管决策中。2020年12月，我国国家药品审评中心发布了《模型引导的药物研发技术指导原则》，提倡在药物研发的全生命周期内积极开展建模与模拟，以支持药品注册申报^[29]^。本文以小分子化药奥希替尼和单克隆抗体药物帕博利珠单抗为例，以问题为导向，阐述了在抗肿瘤药物临床研究中，MIDD方法在药物最佳疗效剂量预测、目标人群E-R关系确定、给药方案优化，以及DDI、特殊人群用药方面发挥的重要作用。随着计算机模拟技术的发展和定量药理研究的深入，可以预见MIDD将在抗肿瘤药物临床研究中发挥越来越重要的作用。
